# Spatial Patterns of Opioid Overdose Mortality Risk Across Urban, Transitional, and Rural Communities Using Satellite-Derived and Socioeconomic Indicators

**DOI:** 10.1007/s11524-026-01081-3

**Published:** 2026-05-11

**Authors:** Gia Barboza-Salerno, Taylor Harrington

**Affiliations:** 1 Colleges of Social Work and Public Health, The Ohio State University, 1735 College Road, Columbus, OH 43210 USA; 2 College of Public Health, The Ohio State University, 352 Cunz Road, Columbus, OH 43210 USA

**Keywords:** Opioid overdose, Nighttime light, Rural, Spatial, Satellite-derived indicators

## Abstract

Geographic differences in drug overdose patterns across the rural–urban continuum are well documented. Few studies utilize remote sensing data to assess environmental and structural factors influencing overdose risk across different geographic scales. We performed a retrospective ecological study analyzing fatal opioid overdoses within census block groups in Cook County, Illinois, from 2018 to 2023. Urbanicity was classified using the Global Human Settlement Layer Model (GHSL-MOD), which employed satellite data to measure built-up intensity, vegetative greenness, and nighttime light intensity (NLI). Environmental indicators were combined with census-based measures of neighborhood deprivation to characterize spatial variation in physical and social conditions. A Bayesian spatiotemporal model was estimated to account for spatial dependence, temporal trends, and environmental exposures, yielding neighborhood-level estimates of overdose risk. Overdose risk exhibited significant spatial clustering and strong associations with both social and environmental factors. Neighborhood disadvantage had a dose–response relationship, with fatal overdose risk in areas with the most deprivation, experiencing over seven times the risk, compared to the least deprived. NLI was strongly associated with increased overdose risk, while vegetative greenness and park access showed no significant protective effects. Increasing trends were detected in rural and transitional zones despite a higher risk in urban centers. Demographic characteristics of overdose victims varied across the county, suggesting potential geographic disparities in risk. The physical and social features of neighborhoods underscore the need for early surveillance and intervention within and outside urban centers. These factors should be incorporated into targeted, place-based strategies to lower opioid-related deaths.

**Supplementary Information:**

The online version contains supplementary material available at 10.1007/s11524-026-01081-3.

## Introduction

Opioid-related overdose is a public health crisis, driven by synthetic opioids like fentanyl, causing record-high overdose rates. Between 1999 and 2020, opioid-related mortality increased by 158% in large metro counties and by 740% in rural areas, with rural Midwestern counties experiencing a staggering 1,600% increase [1]. By 2016, overdose mortality in these rural Midwestern areas was 16 times higher than in urban counterparts [1], underscoring the spread of the opioid crisis beyond urban cores and the growing importance of geographic context [2]. However, standard rural–urban classifications often obscure the overlapping social and environmental conditions that shape opioid-related risk in suburban, peri-urban, and rural communities.

Research has shown that areas classified as rural often face unique risks, including earlier onset of misuse, higher prevalence, and barriers to treatment [3]. Yet structural factors like poverty and education have been shown to predict overdose risk to a larger extent than geography alone. For instance, Pear et al. found that prescription overdose rates were higher in socioeconomically vulnerable communities regardless of setting, while the impact of other risk factors, including low education or unemployment, varied across rural or urban contexts [4]. Similarly, a study in Georgia found that rural counties had higher overdose reversals but that rurality itself was not a consistent predictor once socioeconomic factors were considered [5]. In predominantly White, rural counties, risk is also shaped by isolation and limited healthcare access [6], whereas urban areas are often affected by complex opioid syndemics linked to illicit markets, concentrated disadvantage [7], and heightened law enforcement activity [8]. In these urban settings, vulnerable individuals like younger Black males or unsheltered individuals navigate public drug use in heavily surveilled environments, increasing the risk of arrest and overdose under conditions of racialized policing [9].

Two primary considerations limit existing literature examining opioid-related overdose across different geographical contexts. First, there is no consistent definition of what constitutes “urban” versus “rural.” Most studies rely on classifications from the Census Bureau, OMB, or USDA (e.g., RUCA codes), which focus on population size or commuting patterns. As Palombi et al. note, these definitions often oversimplify heterogeneous contexts and fail to capture conditions in transitional zones, such as peri-urban or exurban communities [10–12]. Roth et al. similarly argue that using population-based definitions to measure rurality obscures meaningful variation in these areas that shape harm-related risk [5]. Second, few studies investigate how these factors interact with built environments across diverse settings. Tempalski et al. call for a socio-built environment framework that integrates infrastructure (e.g., housing density, vacancy, accessibility) with social indicators such as poverty and segregation [13]. However, such frameworks are typically applied in urban settings, with limited use in rural or transitional areas.

Rather than treating areas as binary — rural or urban —satellite indicators capture continuous variation across the landscape, including exurbs, peri-urban zones, and inner-ring suburbs, based on observed land use and development intensity. Built-up areas and NLI emissions reflect infrastructure and human activity, providing more precise spatial insights than traditional classifications such as RUCA codes. Remote sensing data also provide proxies for environmental exposure and land use, revealing features often missed in opioid mortality studies. For example, across multiple studies, NLI correlates with population density, transportation, built-up areas, energy use, and economic activity, highlighting areas with dense infrastructure and nighttime human activity [14–21]. Because outdoor lighting is concentrated where people live, work, and socialize after dark, higher NLI values typically indicate neighborhoods with intense evening and nighttime activity, including mixed-use commercial corridors, entertainment districts, and transportation hubs. Consequently, NLI serves not simply as a measure of illumination but as an indirect indicator of environments in which social interaction, mobility, and economic exchange persist late into the night, conditions that can also facilitate alcohol and drug consumption and the operation of drug markets.

Beyond serving as a proxy for human activity density, exposure to artificial light at night has been increasingly linked to behavioral and biological pathways relevant to substance use and overdose risk. A growing body of research demonstrates that NLI exposure disrupts circadian rhythms and sleep patterns, alters reward processing, and contributes to mood dysregulation and mental health disorders, all of which are associated with elevated vulnerability to substance use and relapse [22–25]. Experimental and epidemiologic evidence further suggests that nocturnal wakefulness is associated with behavioral disinhibition, increased impulsivity, and altered risk perception, conditions that may increase risky substance use and overdose risk, particularly during nighttime hours [26]. In addition, high NLI areas often overlap with other environmental stressors, including pollution, reduced green space, and socioeconomic deprivation, which jointly contribute to chronic stress and mental health vulnerability that can further increase substance use risk [27]. Whereas indicators like impervious surface and the Normalized Difference Vegetation Index (NDVI) are associated with lower stress and overdose risk, dense built environments with limited vegetation often reflect stressful, resource-limited environments [28–31].

Behaviorally, neighborhood structural disadvantage influences overdose risk through various pathways linking socioeconomic hardship to substance use vulnerability. Frameworks that view overdose within the social determinants of health emphasize that structural conditions shape drug initiation, access to care, and overdose vulnerability, positioning deprivation as a core factor driving overdose risk across communities [32]. Numerous studies have shown that areas with higher deprivation face increased rates of opioid prescriptions, drug-related hospitalizations, and overdose deaths [33–35]. Communities with limited economic and social resources expose residents to chronic stress, housing insecurity, unemployment, and fewer opportunities, which are associated with higher psychological distress and increased substance use as a coping strategy [36]. Empirical evidence reveals a dose–response relationship between socioeconomic deprivation and substance-related outcomes, with overdose mortality and drug hospitalizations steadily increasing across deprivation levels, rather than only in the most disadvantaged areas [33, 35]. Similar patterns indicate that employment and economic deprivation are strong predictors of higher opioid use and overdose risk, underscoring how economic insecurity and neighborhood stress influence substance behaviors [34, 37]. Socioeconomic measures such as the Area Deprivation Index (ADI) assess neighborhood-level disadvantage using 17 indicators from the U.S. Census and the American Community Survey across four main domains: income and poverty, employment, education, and housing quality and stability [35, 38, 39]. Together, these studies highlight the importance of integrating satellite data and neighborhood socioeconomic indices to better understand how structural disadvantages and environmental factors jointly contribute to opioid overdose vulnerability [13, 40].

This study uses satellite-derived measures of the built environment to examine fatal opioid overdoses in Cook County, Illinois. In 2022, the county reported nearly 2,000 opioid-related deaths, with fentanyl involved in over 90% of cases [41], and an opioid fatality rate that far exceeds the national average [42, 43]. Although commonly classified as urban, Cook County spans a diverse set of geographies, including dense urban cores, suburban and peri-urban areas, and rural-like settings. Our approach draws on Tempalski et al.’s socio-built environment framework [13] and Neely and Samura’s spatial theory of inequality [44], which conceptualize space as relational, materially produced, and shaped by systems of structural power. Neely and Samura highlight how the built environment can simultaneously foster safety and care while also reinforcing exclusion and surveillance. Their work illustrates how spatial context in areas with different levels of urbanization plays a critical role in shaping overdose risk and access to care for people who use drugs [44]. Accordingly, we ask two central questions: (1) To what extent are satellite-derived features of the built environment associated with fatal opioid overdose risk, after adjusting for social deprivation and urbanicity?; and (2) How do patterns of deprivation and built environment intensity co-locate to form geographic clusters of high overdose mortality, and do these patterns persist over time?

## Methods

### Data

This study employed an ecological approach to examine fatal opioid overdoses across all 2,347 census block groups (CBGs) in Cook County, Illinois, from 2018 to 2023. The dataset was organized as a six-year panel, with annual mortality counts linked to population estimates and covariate data for each CBG. Geographic boundaries for CBGs were standardized to the 2020 TIGER/Line shapefiles and transformed to WGS84 (EPSG:4326) using the *tigris* [45] package.

### Dependent Variable

We downloaded fatal opioid overdose records from the Cook County Medical Examiner’s Case Archive. We filtered the data to retain only those with valid geographic coordinates and death dates from January 1, 2018, to December 31, 2023. To integrate pre-pandemic overdose locations (2018–2019), we employed a spatial interpolation crosswalk to harmonize the data with 2020 geometries using areal weighting. Annual population estimates were retrieved from the American Community Survey (ACS) 5-year data using the *tidycensus* package [46] and merged with CBG shapefiles via unique geographic identifiers. We converted locations of death into spatial features and spatially joined them to 2020 CBG polygons using intersection methods. Annual death counts were then aggregated for each CBG. For block groups with a nonzero population but no observed deaths, zero values were imputed.

### Built Environment

**Fig. 1:**
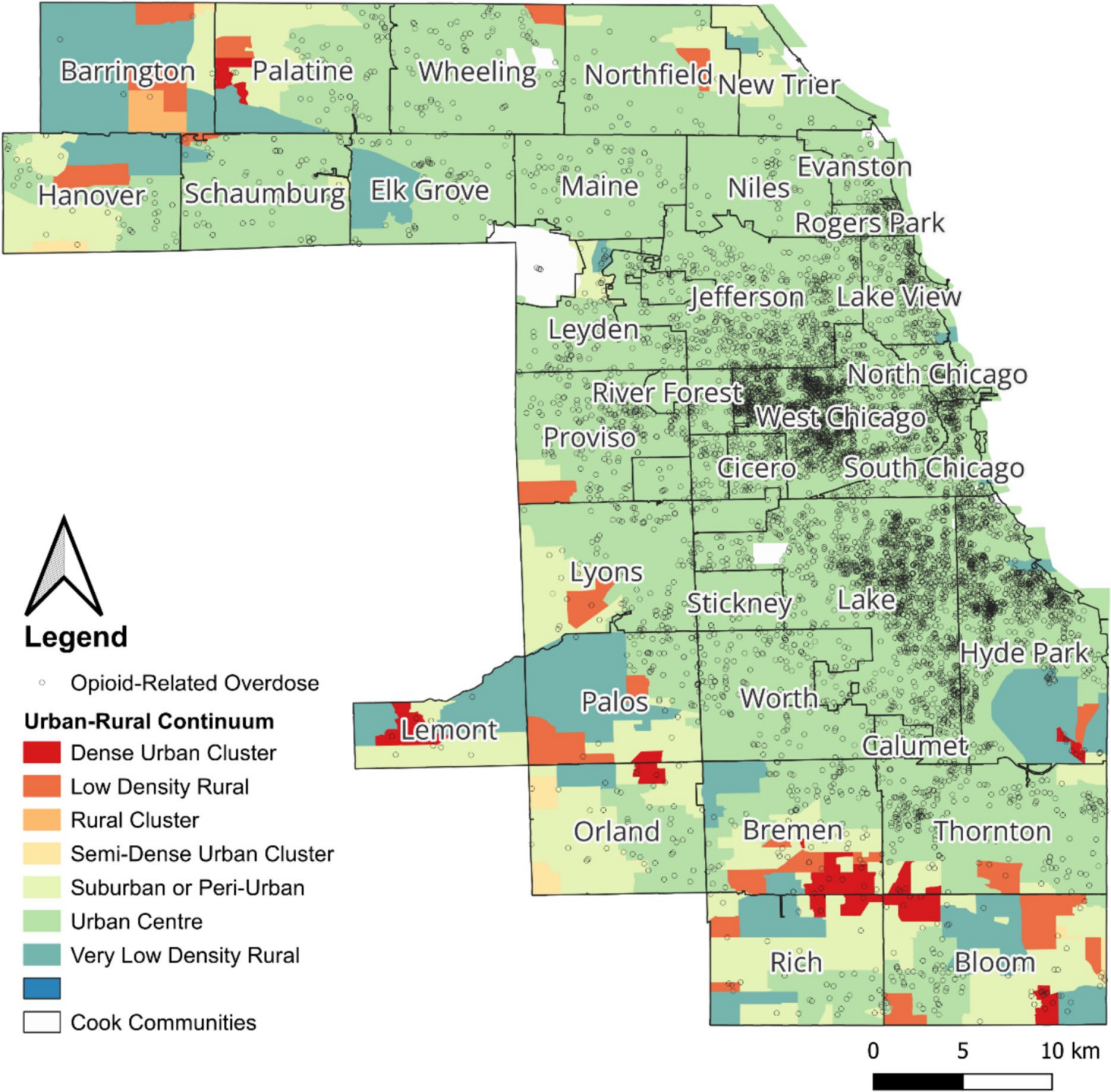
Urban–Rural Continuum and Opioid-Related Overdose Deaths in Cook County, IL (2020–2023). Spatial distribution of opioid-related overdose deaths (black dots) across Cook County census block groups, overlaid with classifications from the Global Human Settlement Layer – Settlement Model (GHSL-SMOD). The urban–rural continuum comprises seven typologies based on built density and settlement structure: Dense Urban Cluster, Semi-Dense Urban Cluster, Urban Centre, Suburban or Peri-Urban, Rural Cluster, Low-Density Rural, and Very Low-Density Rural. Municipal community boundaries (labeled) are shown for reference

Different classifications of urbanization were derived from the 2020 Global Human Settlement Layer Settlement Model (GHSL-SMOD), which categorizes land based on settlement structure and density at a 1 km resolution. Two adjacent raster tiles covering Cook County were mosaicked, and the modal value (i.e., most frequent urban code) within each CBG was extracted using zonal statistics. We collapsed the GHSL-SMOD classes into four broader typologies for interpretation: Urban (dense urban cluster or urban center), Suburban (suburban or pre-urban), Transitional (semi-dense urban cluster), and Rural (low-density or very low-density rural zones). Water-classified areas were excluded due to their small number. Figure 1 shows the urban–rural continuum overlaid onto fatal opioid-related overdoses across the county.

Built Environment Intensity (BEI) was assessed using a normalized raster derived from the GHSL-BUILT dataset, which quantifies the proportion of impervious surfaces (e.g., buildings, pavement) in each 10-m grid cell. The median built-up value within each CBG was extracted using the *exactextractr* package [47]. The index ranges from 0 (entirely undeveloped) to 1 (fully developed), capturing the density of constructed surfaces and serving as a proxy for urbanization, infrastructure, and environmental stressors such as heat exposure and population density.

Nighttime Light Intensity (NLI) was captured from the VIIRS Day/Night Band (DNB) product, which provides stable, cloud-free nighttime radiance measurements from satellite imagery. NLI captured via satellite using the VIIRS reflects both the concentration of human activity and the extent of artificial lighting. These data were rescaled to a 0–1 range and filtered to exclude transient light sources (e.g., fires, aurorae). The median normalized light intensity was calculated for each CBG using zonal extraction.

### Natural Environment

Vegetative Greenness was measured by calculating the Normalized Difference Vegetation Index (NDVI) from Landsat 9 Surface Reflectance bands: the red (Band 4) and near-infrared (Band 5) bands were scaled and combined using the standard NDVI formula:

$$NDVI=\frac{NIR-Red}{NIR+Red}$$

where NIR is near-infrared reflectance, and Red is visible red reflectance. The imagery was filtered to include only cloud-free scenes with less than 10% cloud cover from summer months (June through August). The raster was masked to remove water bodies and cropped to Cook County boundaries. Median NDVI values for each CBG were then extracted and standardized as *z*-scores. Higher NDVI values indicate more abundant and healthier vegetation cover, which has been linked to improved mental health and environmental resilience.

Park Accessibility was measured using the 15-min cumulative park access indicator from TransitCenter's Transit Equity Dashboard. Park accessibility reflects the total park acreage reachable within a 15-min public transit trip from each CBG, accounting for both spatial proximity and transportation infrastructure. Higher values denote greater accessible green space within equitable travel time, making this a more meaningful exposure measure than static distance to the nearest park.

### Social Environment

The ADI, developed by the University of Wisconsin’s Neighborhood Atlas, combines metrics such as income, education, employment, and housing conditions into a composite index [48]. ADI scores for 2016–2020 were merged by GEOID using the *sociome* package [49]. Raw scores were recoded into quintile-based groupings, with Quintile 5 indicating the most deprived neighborhoods. Individual-level incidents were spatially linked to CBG measures, enabling comparisons of age, gender, and race/ethnicity across neighborhood characteristics, including the built environment, deprivation, and vegetation exposure. See Supplementary Tables 1 and 2 for the detailed information regarding data sources and variable construction.

### Statistical Analysis

We computed expected counts for each CBG and year by multiplying their population by the countywide overdose mortality rate for the corresponding year. Standardized mortality ratios (SMRs) were then calculated as the ratio of observed to expected deaths. Expected counts were calculated as:

$${E}_{it}={P}_{it}\times \left(\frac{\sum_{i}{O}_{it}}{\sum_{i}{P}_{it}}\right)$$

where:

- E_it_= expected overdoses in CBG $i$ at year $t$
- P_it_ = population of CBG $i$ at year $t$
- O_it_ = observed overdose count in CBG $i$ at year $t$

Before spatial modeling, fixed-effect Poisson regression models were estimated to explore associations between overdose mortality and neighborhood-level exposures for each year. Covariates were standardized where applicable and entered as main effects. We included a mean-centered year variable defined as the year minus the base year (e.g., ${\mathrm{Year}}_{ct}=t-2018$), ADI quintiles, standardized NDVI, park access, recoded urban classification, BEI, and NLI. All models used a log link and incorporated the log of the population or expected count as an offset.

To evaluate spatial dependence, a spatial contiguity matrix was constructed. Using the *spdep* package [50], a queen contiguity neighbors list was converted into a binary spatial weights matrix where each CBG’s neighbors were assigned equal weights. Moran’s I was computed to assess global spatial autocorrelation in observed overdose rates, and this spatial structure was retained and incorporated into all subsequent Bayesian models. To account for spatial dependence and temporal trends, a Bayesian hierarchical spatiotemporal Poisson model with autoregressive temporal structure was implemented using the *CARBayesST* package [51]. An intrinsic conditional autoregressive (ICAR) prior was specified for the spatial component and temporal autocorrelation was modeled using a first-order autoregressive (AR(1)) process. Letting ${Y}_{it}$ denote the observed number of overdose deaths in CBG_*i*_ at time *t*, and ${E}_{it},$ the expected count, the model assumed that:

$${Y}_{it}\sim {\mathrm{Poisson}}\left({\mu }_{it}\right) {\text{with }} {\mu }_{it}={E}_{it}\cdot {\theta }_{it}$$

The log relative risk was modeled as:

$$\mathrm{log}\left({\theta }_{it}\right)=\alpha +{\beta }_{1}\cdot {\mathrm{Year}}_{ct}+{\beta }_{2}\cdot {\mathrm{ADI}}_{qi}+{\beta }_{3}\cdot {\mathrm{NDVI}}_{i}+{\beta }_{4}\cdot {\mathrm{ParkAcc}}_{i}+{\beta }_{5}\cdot {\mathrm{Urbanicity}}_{i}+{\beta }_{6}\cdot {\mathrm{BuiltEnv}}_{i}+{\beta }_{7}\cdot {\mathrm{Lights}}_{i}+{\phi }_{i}+{\delta }_{it}$$

Here, $\alpha$ is the global intercept, ${\beta }_{k}$ are fixed effects, ${\phi }_{i}$ is the spatially structured random effect capturing spatial dependence via a conditional autoregressive prior, and ${\delta }_{it}$ is an unstructured spatiotemporal random effect. Fixed effects were given diffuse Gaussian priors: ${\beta }_{k}\sim \mathcal{N}\left(0,1000\right)$. Spatial random effects (_i) followed a CAR prior with a precision parameter: ${\tau }_{\phi }\sim {\mathrm{Gamma}}\left(0.5,0.0005\right).$ Spatiotemporal noise followed an exchangeable normal prior with variance: ${\tau }_{\delta }^{-1}\sim {\mathrm{Gamma}}\left(0.5,0.0005\right).$ The temporal autocorrelation parameter was assigned a uniform prior on the interval [0, 1]: $\rho \sim {\mathrm{Uniform}}\left(0,1\right).$ Posterior estimation sampling was conducted using three parallel chains, each running for 20,000 iterations. This included a 5,000 iteration burn-in period and thinning every 10th sample, resulting in 4,500 posterior samples per chain. Model diagnostics included checking for multicollinearity using variance inflation factors (VIF). Model convergence was assessed using trace plots and posterior summaries.

Relative risk (RR) estimates were extracted for each CBG-year combination and mapped using the *ggplot2* package [52]. We descriptively examined the intersection of structural and environmental clustering by mapping the bivariate distribution using the *biscale* package [53]. ADI and NLI were jointly classified using quantile binning (4 × 4 grid), and a custom legend was constructed using *cowplot* [54]*.* Model-derived RR defined high-risk areas as having an RR > 2 and visualized using both single-year and faceted maps. To examine trends in risk over time, both modeled mean relative risk and observed death counts were plotted by year. We used the model-smoothed fitted values from the Bayesian spatiotemporal model to classify CBGs into one of four trend types: sharply increasing, increasing, decreasing, or no change. These trend types were derived by fitting linear and quadratic regressions to the fitted overdose risk over time for each block group and evaluating both the direction and significance of the slope and curvature.

## Results

### Decedent Characteristics

Table 1 displays characteristics of opioid-related overdose deaths by decedent sex. Overall, overdose mortality occurred predominantly among men, who accounted for 7,066 of 9,249 deaths (76.4%), compared with 2,183 deaths among women (23.6%). Male decedents were slightly older than female decedents on average (46.9 vs. 45.4 years; *p* < 0.001), and racial/ethnic composition varied significantly by sex (*p* < 0.001), with males more likely to be Latino/a/e (16.1% vs. 8.7%) and females more likely to be non-Hispanic White (37.5% vs. 31.6%). Manner of death also differed significantly (*p* < 0.001), with suicides representing a slightly larger share of deaths among female decedents (2.8% vs. 0.8%), while accidental overdoses remained more common among males (98.6% vs. 96.0%).

**Table 1:** Characteristics of individuals who died from opioid-related overdose, stratified by sex

Variable	Overall *N* = 9,249^1^	Female *N* = 2,183^1^	Male *N* = 7,066^1^	*p*-value
Age (Mean, SD)	46.5 (13.5)	45.4 (13.6)	46.9 (13.5)	< 0.001
Race/Ethnicity (%)				
Latino/a/e	14.3%	8.7%	16.1%	< 0.001
Non-Hispanic Asian	0.6%	0.8%	0.6%	
Non-Hispanic Black	51.5%	52.5%	51.2%	
Non-Hispanic Indigenous	0.1%	0.1%	0.1%	
Non-Hispanic White	33.0%	37.5%	31.6%	
Other	0.4%	0.4%	0.4%	
Manner of Death (%)				
Accident	98.0%	96.0%	98.6%	< 0.001
Homicide	0.1%	0.1%	0.1%	
Natural	0.3%	0.5%	0.3%	
Suicide	1.2%	2.8%	0.8%	
Undetermined	0.4%	0.6%	0.3%	
Year of Death (%)				0.189
2018	11.7%	12.8%	11.3%	0.007
2019	13.0%	14.3%	12.6%	
2020	18.4%	18.0%	18.6%	
2021	19.1%	19.9%	18.9%	
2022	19.6%	18.7%	19.8%	
2023	18.2%	16.2%	18.8%	
Environmental Indicators (Mean, SD)				
ADI	113.0 (19.6)	113.9 (19.8)	112.7 (19.6)	0.014
Built Environment	0.299 (0.064)	0.292 (0.067)	0.301 (0.064)	< 0.001
Nighttime Light	0.242 (0.1108)	0.228 (0.100)	0.245 (0.100)	< 0.001
NDVI	0.130 (0.086)	0.139 (0.090)	0.128 (0.084)	0.001
Park Access (acres)	60.3 (221.6)	64.0 (169.9)	59.1 (235.3)	0.400
Observed Overdose Counts (Mean, SD)				
2018	0.928 (1.4)	0.885 (1.3)	0.941 (1.4)	0.4
2019	1.06 (1.5)	1.01 (1.4)	1.08 (1.5)	0.9
2020	1.48 (1.9)	1.44 (1.8)	1.50 (1.9)	0.4
2021	1.63 (2.2)	1.58 (2.1)	1.65 (2.2)	0.6
2022	1.64 (2.1)	1.61 (2.0)	1.65 (2.1)	0.2
2023	1.51 (1.8)	1.45 (1.8)	1.53 (1.8)	0.078

Means and standard deviations are reported for continuous variables; percentages are reported for categorical variables. *P*-values reflect results from two-sample t-tests (for continuous variables) or chi-square tests (for categorical variables) comparing female and male decedents, ADI = Area Deprivation Index; NDVI = Normalized Difference Vegetation Index, Environmental indicators reflect the census block group where the decedent’s death occurred, Expected overdose counts were derived using countywide rates adjusted for population size

Environmental exposures varied modestly but significantly by sex. Female decedents were more likely to die in areas with slightly higher socioeconomic deprivation (mean ADI = 113.9 vs. 112.7; *p* = 0.014), lower built-environment intensity (0.292 vs. 0.301; *p* < 0.001), and lower NLI (0.228 vs. 0.245; *p* < 0.001). In contrast, areas associated with female decedents showed slightly higher vegetative greenness as measured by NDVI (0.139 vs. 0.128; *p* = 0.001). Park access did not significantly differ between groups (*p* = 0.400). Observed overdose counts were similar across sexes in each study year, with no statistically significant differences detected from 2018 through 2023. Together, these results indicate that while neighborhood environmental conditions differed modestly across locations associated with male and female decedents, the overall distribution of overdose deaths across CBGs was similar.

**Fig. 2:**
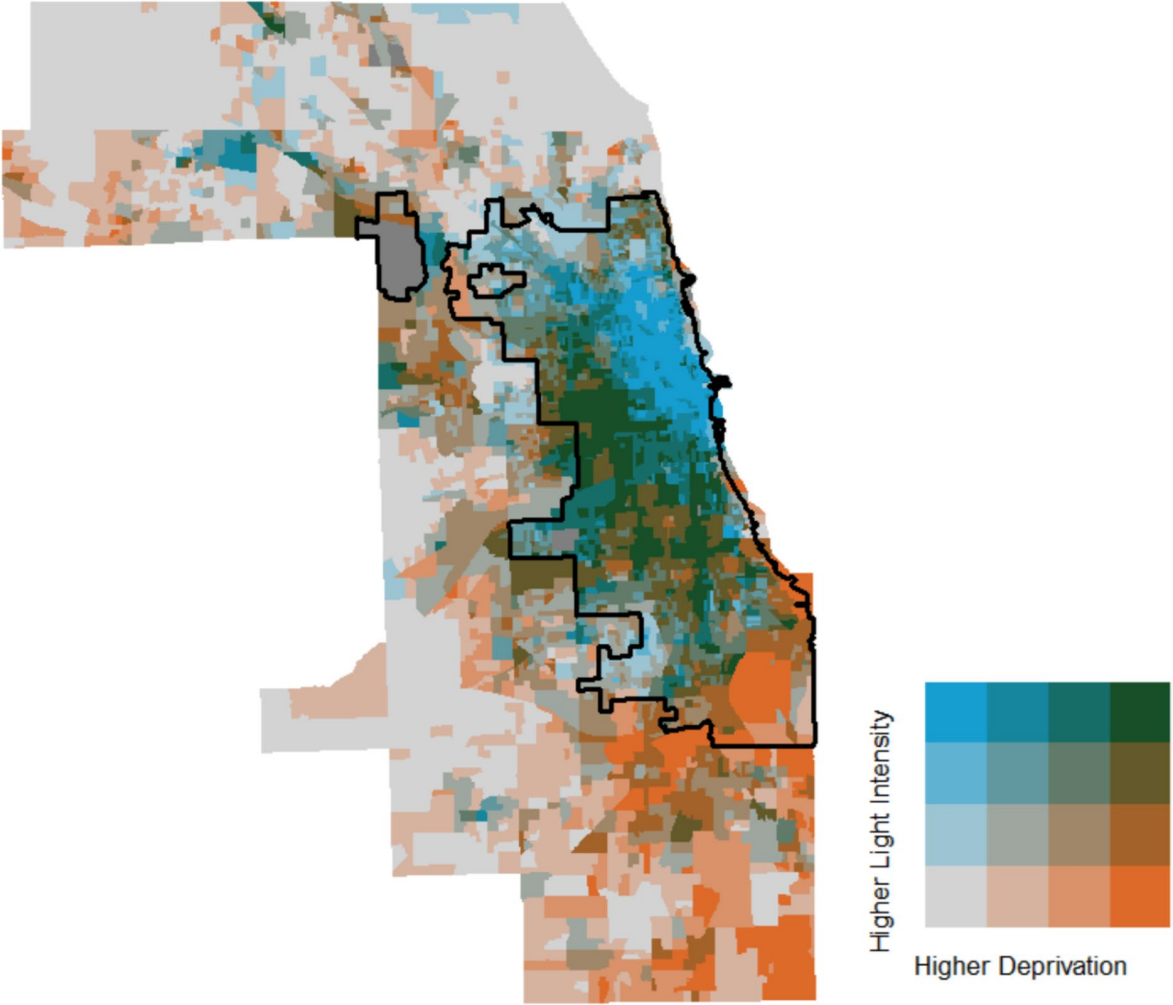
Bivariate choropleth map of Area Deprivation Index (ADI) and Nighttime Light Intensity for Census Block Groups in Cook County, Illinois. Notes. Bivariate map based on the joint distribution of area level deprivation (ADI) and light intensity using quantile-based breaks. Areas are classified as: 1) high deprivation and low light intensity (orange); 2) high deprivation but high light intensity (green); 3) low deprivation and high light intensity (blue), and 4) low deprivation and low light intensity (gray)

Figure 2 illustrates the overlap between ADI and NLI. Areas with high deprivation and bright lights were mainly found in Chicago’s South and West Sides, neighborhoods known for historical disinvestment, but high population density developed.

We examined correlations between environmental and social predictors (see Supplementary Table 3). BEI was significantly and positively correlated with NLI (*r* = 0.496) and negatively correlated with NDVI (*r* = –0.454). NLI and NDVI were strongly inversely related (*r* = –0.577). Park access correlated positively with NDVI (*r* = 0.140) and negatively with built-up area (*r* = –0.280) and NLI (*r* = –0.101). ADI was modestly correlated with BEI (*r* = 0.162, *p* < 0.001) and NLI (*r* = 0.218, *p* < 0.001), and negatively correlated with NDVI (*r* = –0.128, *p* < 0.001) and park access (*r* = –0.062, *p* < 0.001).

Bayesian spatiotemporal Poisson models adjusted for spatial and temporal autocorrelation and included environmental and sociodemographic covariates. VIFs and generalized variance inflation factors (GVIFs) were all well below conventional thresholds (maximum ${G}_{VIF}^{.5df}=1.20$, indicating minimal multicollinearity and stable model estimation; Supplementary Table 4).

**Fig. 3:**
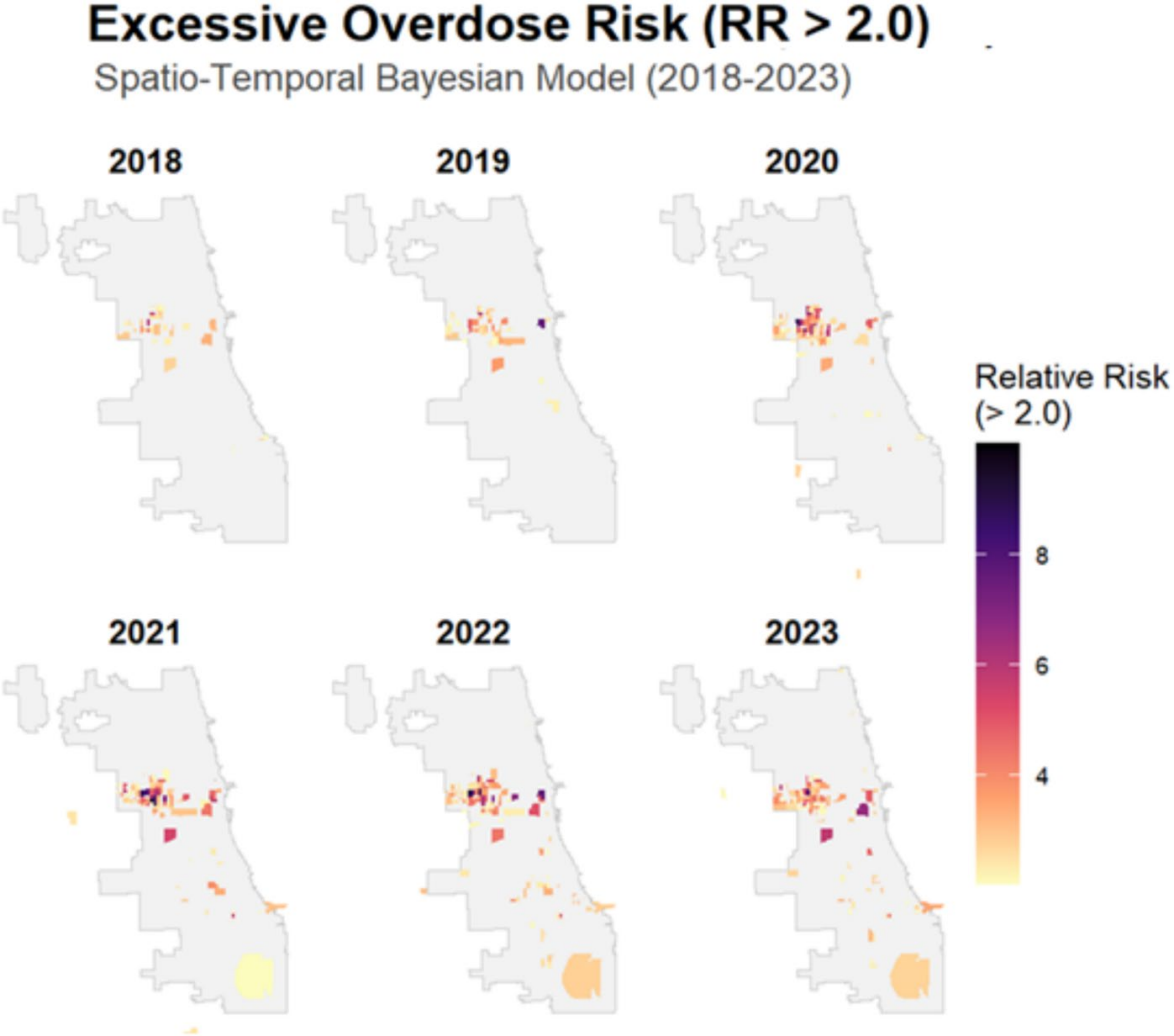
Spatiotemporal Distribution of Opioid Overdose Risk in Cook County, 2018–2023. This figure displays census block groups with estimated opioid overdose relative risk greater than twice the county average (RR > 2.0) for each year from 2018 to 2023, based on the Bayesian spatiotemporal model

The number of high-risk CBGs (RR > 2) increased from 37 in 2018 to 91 in 2021, then to 104 in 2022, before declining slightly to 99 in 2023, suggesting the persistence and spatial spread of overdose risk during and after the COVID-19 pandemic (Fig. 3). Table 2 shows the results from the Bayesian spatiotemporal model. Neighborhood deprivation was strongly and monotonically associated with fatal overdose risk. Compared with the least deprived quintile, CBGs in the second through fifth quintiles had incidence rate ratios (IRRs) of 1.66, 2.43, 2.75, and 4.52, respectively, with all estimates showing narrow credible intervals and strong evidence of a dose–response relationship. In contrast, neither NDVI (IRR = 0.98; 95% credible interval (CrI): 0.94–1.03) nor park access (IRR = 1.01; 95% CrI: 0.96–1.05) were significantly associated with overdose risk in adjusted models.

**Table 2:** Bayesian Spatiotemporal Model Results

Predictor	IRR (95% CrI)	Percent Change (%)
Intercept	0.17 (0.14, 0.21)	–83.1
Year (centered)	1.00 (0.98, 1.01)	–0.26
Area Deprivation Index (ADI) *(ref* = *least deprived)*		
Quintile 2	1.66 (1.47, 1.88)	+ 65.9
Quintile 3	2.43 (2.15, 2.76)	+ 142.5
Quintile 4	2.75 (2.42, 3.14)	+ 175.4
Quintile 5	4.52 (3.97, 5.13)	+ 351.8
NDVI (Vegetative Greenness)	0.98 (0.94, 1.03)	–1.57
Park Access (Acres within CBG)	1.01 (0.96, 1.05)	+ 0.76
Urbanization Category (*ref* = *Urban*)		
Suburban	0.72 (0.57, 0.90)	–28.3
Transitional	0.87 (0.40, 2.18)	–13.5
Rural	1.06 (0.79, 1.38)	+ 5.8
Built Environment Intensity	0.65 (0.37, 1.16)	–34.7
Nighttime Light Intensity	10.24 (7.71, 12.53)	+ 924.0
Spatial Variance (τ^2^)	6.76 (5.62, 8.06)	–
Spatial Autocorrelation (ρS)	2.67 (2.63, 2.69)	–
Temporal Autocorrelation (ρT)	1.09 (1.01, 1.19)	–

Incidence Rate Ratios (IRRs) and 95% Credible Intervals (CrIs) are reported for fixed effects from a Bayesian hierarchical Poisson model with spatial and temporal structure. IRR = exp (Beta); Percent change is calculated as (IRR − 1) × 100

Urbanicity demonstrated mixed effects. CBGs classified as Transitional exhibited lower risk than Urban areas (IRR = 0.87; 95% CrI: 0.40–2.18), while Rural areas showed a non-significant increase in risk (IRR = 1.06; 95% CrI: 0.79–1.38). BEI was also not significantly associated with overdose risk (IRR = 0.65; 95% CrI: 0.37–1.16). In contrast, NLI emerged as a strong positive predictor of risk, with a one-unit increase in standardized light intensity associated with a 10.24-fold increase in overdose mortality (95% CrI: 7.71–12.53). The estimated spatial variance (τ^2^ = 6.76; 95% CrI: 5.62–8.06) and strong residual spatial autocorrelation (ρS = 2.67; 95% CrI: 2.63–2.69) indicate persistent geographic clustering of overdose risk beyond measured covariates.

Regarding temporal trends, mean fitted relative risk increased from 2018 through 2020, remained elevated through 2021 and 2022, and declined slightly in 2023 (Supplementary Fig. 1). However, after accounting for neighborhood socioeconomic and environmental conditions as well as spatial dependence, the year coefficient indicated no consistent countywide linear trend over time (IRR = 1.00; 95% CrI: 0.98–1.01; Table 2). At the same time, the positive temporal autocorrelation parameter indicates that overdose risk exhibited persistence across years (ρT = 1.09; 95% CrI: 1.01–1.19; Table 2), meaning that CBGs with elevated risk in one year were more likely to remain high risk in subsequent years (Fig. 3).

**Fig. 4:**
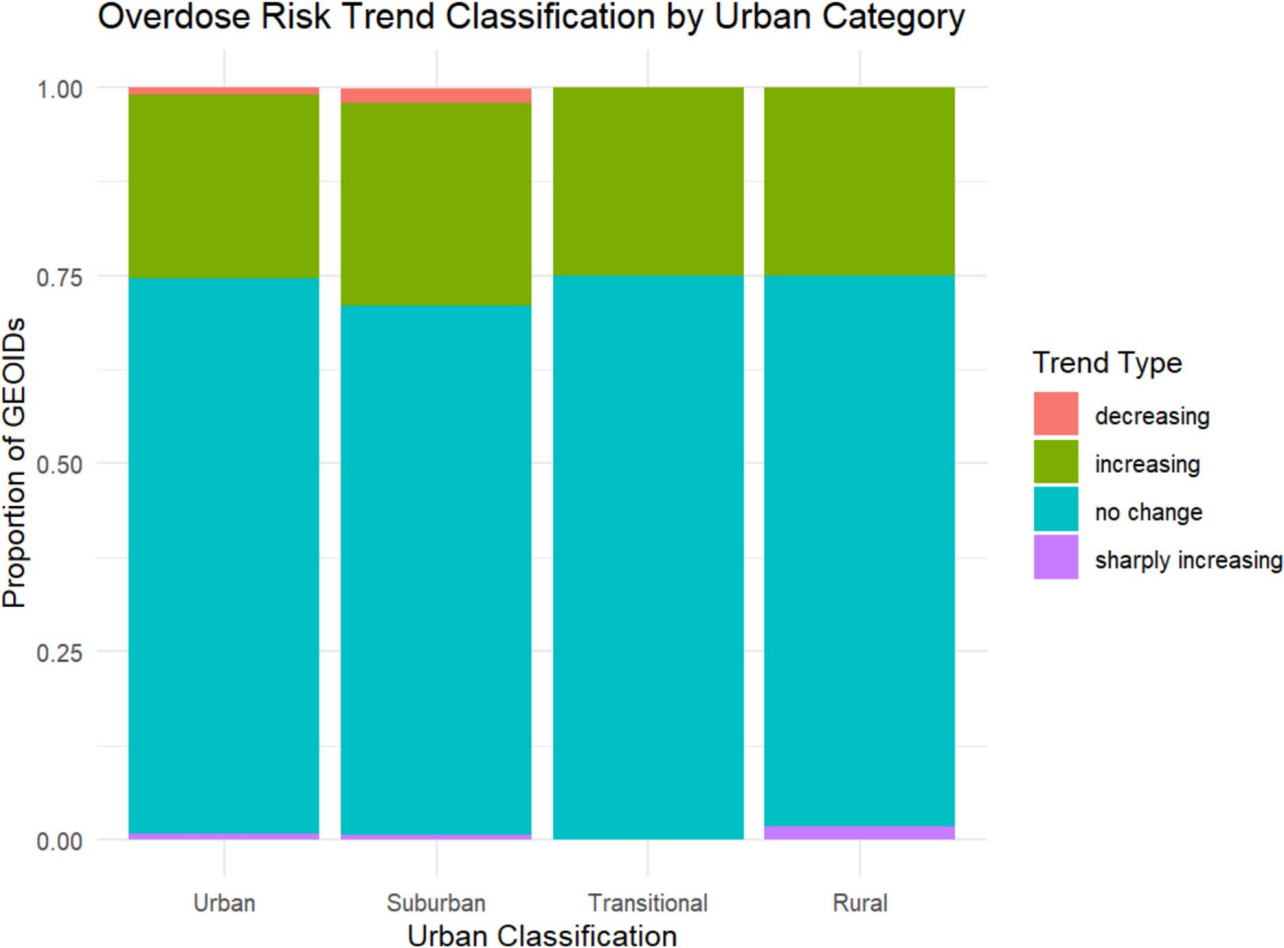
Model-based trend analysis within urban classification. The plot shows the distribution of modeled overdose risk trends across different types of urban environments. The *x*-axis of the plot represents four categories of urbanicity. The *y*-axis shows the proportion of block groups within each category that fall into each of the four trend classifications. Bars are stacked and color-coded by trend type to allow for a direct comparison of the composition of overdose risk trajectories across different geographic contexts

Consistent with these results, the majority of CBGs showed no statistically significant change in overdose trends regardless of urban–rural classification. Specifically, 73.9% of urban block groups showed no change in time trends, compared with 70.4% in suburban, 75.0% in transitional, and 73.2% in rural areas. Approximately one quarter of areas across categories showed increasing overdose trends, including 24.3% of urban, 27.0% of suburban, 25.0% of transitional, and 25.0% of rural block groups. Decreasing trends were rare, occurring only in suburban (1.9%) and urban (1.0%) areas, with no decreases observed in transitional or rural areas. Sharply increasing trends were uncommon overall, occurring in less than 1% of urban and suburban areas and in 1.8% of rural block groups (Fig. 4).

## Discussion

To our knowledge, this is the first study to integrate detailed urban typologies, nighttime light data from the VIIRS sensor, and satellite-derived measures of BEI, alongside ADI-based measures of social disadvantage, to examine how overdose-related deaths vary across diverse socio-environmental contexts. Our findings align with a small but growing body of research emphasizing the importance of considering both structural disadvantage and physical environmental characteristics in understanding the opioid overdose crisis [40]. Similar to Williams et al. [40], who analyzed 565 municipalities across New Jersey to capture meaningful variation in infrastructure, social conditions, and service environments, our study demonstrated that environmental conditions shape overdose risk across diverse neighborhood contexts. Rather than constructing a composite index, however, we focused on isolating the independent and categorical effects of specific environmental exposures and examined how overdose risk varies across discrete urban typologies.

We found persistent geographic and temporal clustering of overdose risk, underscoring the importance of spatially explicit models for identifying trends and informing prevention efforts. The number of CBGs with overdose risk exceeding 150% of the county average increased, mainly in highly urbanized Chicago areas on the South and West Sides, regions historically affected by disinvestment, racialized poverty, and organized abandonment [55]. Model-estimated risk increased between 2018 and 2020, the period preceding the onset of the COVID-19 pandemic, although the overall trend over the period was insignificant. Pandemic-related disruptions likely contributed to the increases in overdose risk observed during the study period, particularly in transitional and highly disadvantaged communities where treatment and social supports were already limited prior to COVID-19. Around the globe, pandemic containment measures coincided with reductions in access to substance use disorder treatment [56], harm reduction services [56, 57], and chronic pain management [58], while clinic closures, reduced in-person services, and regulatory barriers to care resulted in challenges for treatment initiation and continuation. In the United States, the lack of federal policy to address treatment expansion left hundreds of thousands of people without access to treatment [59]. These service interruptions increased relapse risk, interrupted opioid agonist therapy, and pushed some individuals toward illicit and often more dangerous drug supplies, thereby increasing overdose risk [60–62]. At the same time, longstanding disparities in healthcare infrastructure meant that communities already facing socioeconomic disadvantage and limited treatment availability experienced disproportionate barriers to care during the pandemic, compounding preexisting vulnerabilities in areas with fewer healthcare and recovery resources, regardless of stay-at-home mandates [61, 63–66]. Another explanation for the significant increase in opioid-related deaths in some areas is disruptions to drug supply chains resulting from COVID-19 mitigation policies, which reduced access to drugs and may have led to the use of “riskier substance use” [67] [p. 5].

As with previous work [68, 69], our findings have direct public health relevance for guiding where resources and prevention efforts may be most effectively targeted and for supporting the monitoring and forecasting of substance use–related harms. Our study highlights the importance of identifying high-risk and transitional zones to guide local strategies like expanding syringe programs or deploying outreach teams in underserved overdose hotspots lacking harm reduction infrastructure. In these areas, new efforts to guide opioid-related interventions may be needed, such as enhanced opioid education, naloxone distribution, and increased access to medication for opioid-use disorder (MOUD), including mobile treatment units and community naloxone distribution hubs in more remote areas [69]. Improvements to neighborhood infrastructure, such as enhanced lighting and increased access to green space, may also help improve service accessibility in areas with fewer established resources. On the other hand, in stable regions, current strategies could be strengthened if the local infrastructure of civic and social services is adequate [68]. Future research would benefit from incorporating distinctions among urban, suburban, peri-urban, and rural contexts to better understand how environmental and social factors interact across the urban–rural continuum.

Our findings support a growing body of research identifying NLI as a chronic environmental stressor associated with a wide range of adverse health outcomes [16, 70], including sleep disorders [71], preterm birth [14], gestational diabetes [72], colorectal cancer [73], and mood disorders. NLI has also been linked to depressive symptoms, anxiety, and suicidal ideation or attempt [71, 74]. These effects are attributed to NLI’s disruption of sleep cycles and circadian rhythms, which reduces melatonin production and impairs immune and hormonal function. Such circadian disruptions have, in turn, been associated with increased vulnerability to substance use and addiction [22]. Importantly, NLI also reflects the presence of other environmental stressors that contribute to poor health. For example, areas with high NLI exposure tend to have less green space, more air pollution, concentrated poverty, and greater area deprivation [70], conditions that are independently linked to poor mental health [70] and increased risk for substance misuse [75]. These overlapping exposures suggest that NLI contributes to overdose vulnerability both through direct biological mechanisms and as an indicator of broader structural disadvantage.

Regarding area deprivation, we found a dose–response relationship between opioid-related overdose and ADI consistent with past studies [65]. NLI contributed to geographic disparities in overdose deaths in urban areas of Chicago experiencing concentrated structural disadvantage. These findings align with prior research showing that NLI exposure is significantly higher in the most socially vulnerable neighborhoods, disproportionately affecting racially and ethnically minoritized communities [74]. We also note that the least deprived neighborhoods exhibited lower levels of NLI, despite their dense population and high economic activity. At a minimum, our results contribute to a growing body of evidence that identifies NLI exposure not only as a public health concern but also as a potential marker of environmental injustice [74].

We also observed that opioid-related fatality rates were significantly lower in transitional areas, which do not fit neatly into entirely urban or rural categories. However, these areas experienced the sharpest increases in overdose risk for the period we considered, which overlaps with the COVID-19 pandemic. This suggests that areas associated with lower risk may represent emerging hotspots with trends that increase over time. By contrast, suburban or peri-urban areas exhibited the most stable overdose trajectories, with over 96% of block groups showing no significant change across the study period. This relative stability may reflect a more substantial presence of protective factors in these areas [76].

Consistent with prior research [77], we found no significant association between opioid-related mortality and measures of vegetative greenness (e.g., NDVI) or park access. This suggests that, at least at the spatial resolution and form captured in this study, the availability of green space may not confer a measurable protective effect against overdose. Additionally, our satellite-derived measure of BEI, which reflects infrastructure density and land-use patterns, was not significantly associated with opioid mortality after adjusting for other covariates.

Although examination of sex differences and environmental exposures was not the primary aim of this study, several descriptive patterns are worth noting. Overdose mortality occurred predominantly among men, who accounted for three out of every four deaths. Regardless of the decedent’s sex at birth, the overwhelming majority were classified as accidental overdoses, accounting for 96.0% of female deaths and 98.6% of male deaths, while suicides represented only a small share. This pattern is consistent with broader national trends indicating that overdose mortality has historically been higher among men, although the gender gap has narrowed over time as overdose deaths among women have increased, particularly during the fentanyl era [78–80].

At the same time, female decedents were somewhat more likely to reside in areas characterized by higher socioeconomic deprivation, lower BEI, reduced NLI exposure, and diminished vegetative greenness than those associated with male decedents. This pattern aligns with research on gender and substance use, which highlights the disproportionate stigma, trauma exposure, and structural barriers that women face in accessing care [81]. There is also evidence that females may be more biologically sensitive to circadian disruption and socially vulnerable to environmental neglect, both of which may increase overdose risk in underrecognized ways. While prior studies have generally not identified significant sex-specific interactions with NLI exposure for outcomes such as preterm birth or gestational diabetes [14, 15], underlying physiological or contextual differences may still shape how environmental exposures relate to health across populations. Because examination of sex-specific environmental effects was beyond the scope of the present study, future research would benefit from evaluating how overdose mortality patterns differ across built and environmental contexts by sex, as our findings suggest a potentially important dimension of overdose vulnerability that warrants further investigation.

This study has several limitations that should be acknowledged. First, the analysis is limited to Cook County, Illinois, which includes the city of Chicago, an area characterized by distinct patterns of segregation, infrastructure, and access to services. As such, the findings may not be generalizable to regions with different geographic, demographic, or policy contexts. Second, although we modeled time-varying estimates of overdose risk, we did not incorporate time-varying covariates. Environmental exposures are shaped by both natural and human factors that vary across time and space [82]. Therefore, future research should investigate how temporal changes in social and ecological conditions may influence overdose patterns across levels of urbanization. Third, the study period was relatively short and coincided with the COVID-19 pandemic. Despite studies finding no association between social distancing and opioid-related overdose in Cook County [65], the unique context may limit the detection of long-term or typical trends, as the pandemic introduced widespread disruptions in healthcare access, social services, and substance use patterns [83–86]. Finally, Cook County lacks a balanced representation of rural, suburban, and urban areas, restricting our ability to assess overdose risk across the full urban–rural continuum. Future studies should apply these methods in regions with greater spatial diversity to more fully examine how social and environmental conditions interact across varying geographic contexts to shape overdose vulnerability.

## Conclusion

Proactive public health prevention should consider both the physical and social environments and the demographic characteristics of the population residing there. The integration of remote sensing data provides new insight into how features of urban form and human activity influence opioid-related health outcomes beyond what is captured by traditional socioeconomic indicators alone. Since remote sensing data sources are routinely updated and publicly available, our framework can be used by local governments and health departments to monitor changing neighborhood conditions, validate emerging overdose hotspots with community partners, and support the identification and local validation of emerging overdose risk in transitional areas by capturing patterns often missed by traditional definitions of rurality and urbanicity.

## Supplementary Information

Below is the link to the electronic supplementary material.

ESM 1 (DOCX 1.47 MB)

## Data Availability

Links to all data sources are provided in Supplementary Table 1.
