# Supplementary material for: Spatial Patterns of Opioid Overdose Mortality Risk Across Urban, Transitional, and Rural Communities Using Satellite-Derived and Socioeconomic Indicators
Source: J Urban Health. 2026 May 11;103(3):557–73. doi: 10.1007/s11524-026-01081-3 (PMC13315396; doi:10.1007/s11524-026-01081-3)
Supplement: Supplementary file 1 — (DOCX 1.47 MB) [file 11524_2026_1081_MOESM1_ESM.docx]

| **Supplementary Table 1.** Data Sources and Variables | | |
| --- | --- | --- |
| Data Source | Variable | URL |
| USGS Earth Explorer | NDVI (Landsat 9 surface reflectance bands) | <https://earthexplorer.usgs.gov/> |
| Copernicus GHS-POP | Population density (inh/km²) | <https://human-settlement.emergency.copernicus.eu/download.php?ds=pop> |
| Copernicus GHS-BUILT-S | Built-up area (m² built per km² cell) | <https://human-settlement.emergency.copernicus.eu/download.php?ds=built-s> |
| Earth Observation Group (Payne Institute) | Nighttime lights radiance (VIIRS DNB, nW/cm²/sr) | <https://eogdata.mines.edu/products/vnl/> |
| University of Wisconsin Neighborhood Atlas | Area Deprivation Index (ADI) | <https://www.neighborhoodatlas.medicine.wisc.edu/> |
| U.S. Census Bureau (via tidycensus / API) | Block-group total population (Decennial & ACS5) | <https://api.census.gov/data.html> |
| Chicago Data Portal (Socrata) | Non-fatal firearm incidents (People Shot, Chicago) | <https://data.cityofchicago.org/Public-Safety/People-Shot-Chicago/ijzp-q8t2> |

| **Supplementary Table 2.** Data Measures & Definitions | | |
| --- | --- | --- |
| Measure | Definition / Description | Conceptual Distinction |
| Year | Calendar year of observation, modeled as a continuous variable. | Captures time trend in overdose risk; distinct from place-based structural or environmental characteristics. |
| ADI Quintile | Area Deprivation Index grouped into quintiles. Higher quintiles indicate greater socioeconomic disadvantage based on factors like poverty, unemployment, and education. | Reflects structural social disadvantage, distinct from environmental or urban form measures. |
| NDVI (Vegetation) | Standardized mean Normalized Difference Vegetation Index. Higher values indicate more vegetation or green space. | Captures natural environment quality, independent of built structures or social disadvantage. |
| Park Accessibility | Park acreage reachable with 15-minute drive time; higher levels = more access to parks | Captures greater accessibility to public parks |
| Urban Category | Recorded measure of the urban-rural continuum to include 1) Rural: Very Low Density and Low Density Rural areas; sparse settlement, minimal development. – 2) Transitional: Semi-Dense Urban Cluster, 3) Suburban: suburban or Peri-Urban areas; moderate development, mixed character. and 4) Urban: Dense Urban Cluster and Urban Centre areas; highly developed, concentrated urban core. - | Differentiates settlement form and density. |
| Built Environment | Standardized index reflecting built infrastructure intensity (e.g., building density, road network). | Reflects physical development intensity |
| Light Environment | Standardized measure of nighttime light intensity from satellite imagery. Higher values reflect greater artificial light and human activity. | Proxy for human activity and development, complements built environment measures but captures different aspects of urban intensity. |
| Offset (Population) | Log of total population, included to model rates rather than raw counts. | Adjusts for underlying population size; necessary to interpret model results as rates. |

| **Supplementary Table 3**. Correlations between socio-built environmental features | | | |
| --- | --- | --- | --- |
| Variable 1 | Variable 2 | Pearson’s Correlation | *p*-value |
| Area Deprivation Index | Built-up Area Intensity | 0.162 | < 0.001 |
| Area Deprivation Index | Nighttime Light Intensity | 0.218 | < 0.001 |
| Built-up Area Intensity | Nighttime Light Intensity | 0.496 | < 0.001 |
| Area Deprivation Index | Vegetative Greenness (NDVI) | -0.128 | < 0.001 |
| Built-up Area Intensity | Vegetative Greenness (NDVI) | -0.454 | < 0.001 |
| Nighttime Light Intensity | Vegetative Greenness (NDVI) | -0.577 | < 0.001 |
| Area Deprivation Index | Park Access (Acres within CBG) | -0.062 | < 0.001 |
| Built-up Area Intensity | Park Access (Acres within CBG) | -0.280 | < 0.001 |
| Nighttime Light Intensity | Park Access (Acres within CBG) | -0.101 | < 0.001 |
| Vegetative Greenness (NDVI) | Park Access (Acres within CBG) | 0.140 | < 0.001 |

Notes. Pearson’s correlation coefficients (*r*) for pairwise associations between key environmental and social variables. All *p*-values are statistically significant (i.e., < 0.05). NDVI = Normalized Difference Vegetation Index; CBG = Census Block Group

| **Supplementary Table 4**. Multicollinearity Diagnostics | | | |
| --- | --- | --- | --- |
|  | GVIF | df | GVIF^(1/(2*Df)) |
| Year | 1.000 | 1.000 | 1.000 |
| ADI | 1.080 | 4.000 | 1.010 |
| NDVI | 1.444 | 1.000 | 1.202 |
| Park Access | 1.248 | 1.000 | 1.117 |
| Urbanicity | 1.542 | 7.000 | 1.031 |
| BEE | 1.399 | 1.000 | 1.183 |
| NLI | 1.408 | 1.000 | 1.187 |

Notes. Generalized Variance Inflation Factors (GVIFs) are reported along with degrees of freedom (*df*) and adjusted GVIFs [GVIF^(1/(2*df))] for covariates included in the fully adjusted Bayesian model. Values below 2 indicate minimal multicollinearity.


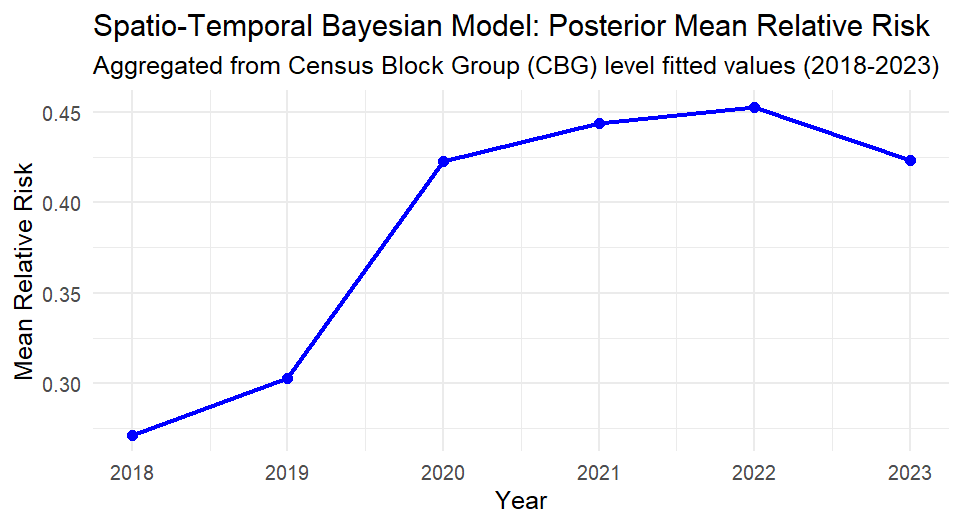


**Supplementary Figure 1.** Mean Posterior Relative Risk of Opioid-Related Overdose Mortality, 2018–2023. This figure displays the mean posterior relative risk of opioid-related overdose mortality estimated from the Bayesian spatiotemporal model and aggregated across census block groups in Cook County. Model-estimated relative risk increased from 2018 through 2020, remained elevated during 2021 and 2022, and declined slightly in 2023. Although the centered year coefficient was not statistically significant after accounting for neighborhood socioeconomic, built environmental, and spatial factors, indicating no uniform countywide linear time trend, the temporal autocorrelation parameter was significant, demonstrating persistence in overdose risk over time. This suggests that areas with elevated overdose risk in one year tend to remain high risk in subsequent years, and that observed temporal changes reflect sustained neighborhood-level risk patterns rather than a consistent countywide trend.
